# Nursing Interventions to Reduce the Burden of Informal Caregivers of People With Chronic Illness: A Narrative Review

**DOI:** 10.7759/cureus.108843

**Published:** 2026-05-14

**Authors:** Patrícia P Oliveira, Óscar M Ferreira

**Affiliations:** 1 Medical-Surgical Nursing, Lisbon School of Nursing, University of Lisbon, Lisbon, PRT; 2 Nursing Fundamentals, Lisbon School of Nursing, University of Lisbon, Lisbon, PRT

**Keywords:** burden caregiver, chronic illness care, home care, informal caregiving, nursing interventions

## Abstract

Increasing chronic pathology’s prevalence and the paradigm shift in healthcare services have created the necessity of a central figure in home care management: the informal caregiver. This role is associated with significant psychological and physical strain. Despite increased recognition, healthcare professionals often lack a comprehensive understanding of the impact of caregiving on these individuals. This article aims to analyze the determinants of informal caregiver burnout and identify nursing interventions effective in burden mitigation. A narrative literature review was conducted across the MEDLINE Ultimate and CINAHL Ultimate databases, spanning a 10-year period. The search included studies in Portuguese, English, and Spanish. A final sample of 10 articles was selected for analysis. Several variables associated with both the caregiver and the care recipient were identified as significant burnout determinants. These include the recipient’s advanced age and functional dependency and the caregiver’s age, gender, and marital status. The most effective interventions were categorized as psychoeducational, multicomponent, psychotherapeutic-counseling, and technology-based. The implementation of structured nursing interventions, with the support of specialized clinical expertise, is essential to ensuring that caregivers receive high-quality health support and resources.

## Introduction and background

A chronic illness diagnosis within a family often predicts the emergence of an informal caregiver, who assumes a pivotal role in managing the health-illness continuum of the patient. The informal caregiver is an individual, whether or not related by family ties, who takes on the responsibility of providing care to another person without receiving any income [1].

Statistical data from 2024, published by the Portuguese National Institute of Statistics in 2025, indicates that 42.3% of the population 15yo+ suffered from at least one chronic condition. Notably, 33.4% of these individuals reported limitations in performing activities of daily living. This prevalence is significantly higher among the geriatric population (65+), where seven out of ten individuals present with chronic comorbidities. These data place Portugal as the third-highest nation within the Organization for Economic Cooperation and Development (OECD) regarding chronic disease prevalence, trailing only Finland and Estonia [2].

In alignment with the 2023-2026 governmental program, Action Plan for Active and Healthy Aging, the primary drivers of disability adjusted life years (DALYs) are cardiovascular, musculoskeletal, respiratory, oncological, endocrine, and mental health disorders. These conditions are major contributors to functional dependency [3]. Data from July 2025 indicates there are approximately 18,000 officially recognized informal caregivers in Portugal, a 19% year-over-year increase. This demographic is predominantly female with a mean age of 60 years [4].

The conjunction of both the abovementioned data as well as the current organizational structure of health services, characterized by shorter hospital stays, suggests a paradigm shift where the primary responsibility for care falls upon informal caregivers. Consequently, health systems have a fundamental duty to support this unprepared population.

However, a 2021 study by the Movement to Care for Informal Caregivers revealed significant gaps in support: 81% of caregivers deemed existing services and support insufficient, while substantial proportions reported a lack of personal time (39%) and social isolation (25%). On a scale from zero (none) to ten (extreme), respondents reported high levels of physical (7.96) and emotional (8.44) exhaustion, underscoring the deleterious impact of domiciliary caregiving on life quality [5].

The literature corroborates these findings, establishing that caregiving often leads to a negative impact on the quality of life and health of family caregivers. This is a multidimensional problem involving emotional distress, anxiety, and deterioration of physical and social well-being. These negative effects are frequently referred to and described as overload, tension/burnout, and stress [6].

Informal caregiver burden is defined as a negative emotion or stress experienced by informal caregivers that arises from providing care to another. It is a negative outcome of the caregiving experience, exacerbated by the multiple roles and responsibilities that informal caregivers must fulfil [7]. In a chronic disease, the caregiving context is an outcome exacerbated by the accumulation of roles, socioeconomic variables and individual factors from both caregiver and care receiver [8]. The caregiver’s responsibilities are exhaustive, ranging from clinical tasks (managing medical devices and treatment regimens) to logistical and emotional support [9,10]. Chronic exposure to these demands can trigger isolation, emotional and depressive symptoms, and physiological manifestations, including hypertension, gastrointestinal distress, and musculoskeletal disorders [11]. Despite the legislative framework provided by the Informal Caregiver Statute [12], healthcare professionals remain unaware of the impact that caregiving has on the informal caregiver in Portugal.

There is a notable absence of recent data among the Portuguese population as well as internal institutional policies aimed at mitigating this issue, despite the availability of validated assessment tools such as the Zarit Scale and the Informal Caregiver Burden Assessment Questionnaire. Thus, it is imperative for healthcare professionals, especially nurses, to refine their technical and pedagogical competencies to provide individual-oriented health education. Such interventions are vital for fostering adaptive responses and reducing the incidence of burden [13].

Given the scarcity of research specifically focusing on nursing-led strategies for this population, this narrative review seeks to synthesize the determinants of caregiver burden and identify evidence-based nursing interventions. This article was previously presented as a poster at the Webinar "Equidade, Segurança e Sustentabilidade: Uma Enfermagem Médico-Cirúrgica centrada nas pessoas" on February 4, 2026.

## Review

Methods

This narrative review synthesizes heterogeneous evidence to identify determinants of informal caregiver burden and evaluate relevant nursing interventions. A comprehensive search was conducted via the EBSCOhost platform (MEDLINE Ultimate and CINAHL Ultimate) and Google Scholar for gray literature. Search strings employed DeCS/MeSH descriptors, including "Caregiver Burden," "Informal Caregiver," "Chronic Disease," and "Nursing Interventions," and a 10-year time frame was chosen, given the scarcity of relevant research on the topic. The articles were in Portuguese, English, or Spanish and addressed the burden of informal caregivers of people with chronic diseases and nursing interventions focused on mitigating the burden of informal caregivers.

The eligibility criteria were as follows: inclusion criteria included studies addressing the burden on informal caregivers of people with chronic illness and/or describing/evaluating nursing interventions aimed at caregivers with explicit outcomes regarding the burden. Exclusion criteria included articles without full text, duplicates, editorials, letters to the editor, conference abstracts, studies not focused on informal caregivers, or studies lacking data relevant to the objectives.

The selection process involved a primary screening of titles and abstracts followed by a critical full-text appraisal. Ten articles met the eligibility criteria. Data extraction was standardized using a matrix to categorize authors, methodology, sample, burden assessment instrument, determinants of informal caregiver burden, nursing interventions implemented, and effects on burden. Table 1 below summarizes these articles.

**Table 1:** Findings of the 10 articles included in the review ICB: informal caregiver burden; IC: informal caregiver

Reference	Study design	Objective	Sample Size	Study population	Key findings
Choi et al. (2024) [6]	Systematic review of observational studies	Identify factors associated with ICB	18 studies	IC of older adults (≥65 years) with chronic disease	The main determinants associated with the care recipient were age (ICB increases with advancing age), functional dependence (associated with activities of daily living, namely, eating and using the toilet), chronic comorbidities, behavioral issues, and neuropsychiatric conditions. The most frequent factors associated with the caregiver were as follows: gender (female caregivers are associated with higher ICB) and age (a nonlinear relationship). Higher levels are reported among both younger and older caregivers; kinship with care recipient (children and spouses reported higher levels); education (both at lower and higher levels of scholarity reported higher levels of ICB); the duration and intensity of caregiving (a direct association with ICB); health status (multiple comorbidities, or complex treatment regimens exhibited higher ICB); employment (professional activity was described as a protective factor)
Ali et al. (2023) [14]	Cross-sectional study	Understand ICB among cancer patients, as well as the factors associated with it	347	IC of outpatients with cancer undergoing follow-up/treatment at an oncology center	66.6% reported high levels of the caregiving burden inventory. Perceived social support was generally moderate to high. The more significant determinants were female gender (female caregivers were twice as likely to develop burden); daily duration of care (more than 8 hours of daily care increased the likelihood of ICB approximately three times); previous hospitalization (IC of patients with a prior history of hospitalization were twice as likely to develop ICB); sleep deprivation (caregivers with <7 hours per day doubled the risk of burden)
Rafiei et al. (2025) [15]	Descriptive, quantitative, cross-sectional study with a correlational approach.	Examine ICB among people with ostomies	250	IC of patients with intestinal ostomies	The average ICB score was 85.5, corresponding to severe burden. The most associated determinants were marital status (married caregivers exhibited significantly higher ICB levels); caregivers’ spirituality (higher levels of spiritual well-being are associated with lower levels of ICB)
Liu et al. (2023) [13]	Qualitative study (semi-structured interviews)	Understand the feelings and experiences of IC of patients with Crohn’s disease	11	IC of patients with Crohn’s disease who have a temporary ostomy	Five main themes were identified that characterize the experience of IC of patients with Crohn’s disease with temporary ostomy: negative psychological experiences (guilt, anxiety, fear, and shame) are associated with the social stigma of the disease. Perception of ICB (emotional exhaustion associated with the chronic nature of the disease, physical fatigue from continuous vigilance, the economic impact of the disease, and the need to reorganize personal and professional life). Uncertainty about the future (associated with ostomy reversion and the unpredictable nature of the disease). Sense of benefit associated with the disease (a paradoxical process of positive adaptation, including personal growth, increased empathy, a reevaluation of values, and a strengthened sense of fulfillment). Inadequacy of the support system (limited educational resources, difficulties in accessing professional support, and the absence of structured follow-up).
Kaynar & Vural (2018) [10]	Prospective, cross-sectional, and descriptive study	Investigate the perception of ICB and associated reactions among caregivers	162	Family caregivers of patients undergoing surgery for colorectal cancer	66.7% of the patients had a stoma. Significant associated determinants in this study: presence of a stoma, associated with higher levels of ICB; age, older caregivers exhibited higher levels of ICB; duration of caregiving, increased time spent on caregiving showed a moderate positive correlation with burden; socioeconomic and educational factors, higher ICB levels were observed in caregivers with lower education and incomes; absence of support systems, increased ICB; kinship proximity, spouses exhibit higher levels of burden; postprocedural, the postoperative period, exhibited increased levels of burden
Maguire et al. (2016) [16]	Analytical cross-sectional study	Analyze predictors of ICB in colorectal cancer survivors.	153	IC of colorectal cancer survivors	Patient characteristics constitute the main determinant of burden. Other identifiable determinants: self-perception of health, associated with higher levels of ICB; presence of a stoma; care-related activities/time spent providing care. Caregiver determinants had less impact. Nevertheless, of note: comorbidities; parenthood; age, younger caregivers exhibited higher levels of financial burden; economic costs
Bernal et al.(2018) [17]	Quasi-experimental	Evaluate the implementation of a nursing intervention on the well-being of IC of people requiring home care	72	IC of patients with chronic disease	Intervention grounded in Swanson’s theory. Step 1: Understand the dyad reality and needs. Step 2: Active listening and emotional support to caregivers. Step 3: Reinforce the caregiver’s self-confidence. Step 4: Promote the patient’s autonomy. Step 5: Develop home care skills and follow-up support assurance. The impact of Individualized educational sessions and structured support was: Significant improvement of IC´s well-being; Reduction in negative emotional factors regarding the family member’s health condition; Improved understanding of the health-illness process; Improved perception of the caregiver’s role; Heightened self-confidence; Emotional expression improvement; Enhancement in the dyad relationship; Improved care-related knowledge; Promotion of autonomy in caregiving; Enhanced situational adjustment and adaptation
Lopes & Arco (2019) [18]	Integrative Literature Review	Review interventions that prevent/reduce ICB in dependent older adults in the community, based on Orem’s Self-Care Deficit Theory	9 articles	IC of dependent older adults in the community	The evidence categorized effective interventions in four domains. Psychoeducational interventions: These interventions demonstrate a positive impact on the IC´s self-efficacy, role adjustment, and emotional management. Psychotherapy/counseling interventions: contributing to the reduction of anxiety, stress, and emotional overload symptoms. Multicomponent interventions integrating multiple strategies. Provided more consistent and sustained effects in burden reduction. Technology-based interventions have proven effective, particularly when complementary to other interventions. The effectiveness of interventions is associated with the presence of specific structural characteristics, namely, prolonged duration (6 to 12 months); involvement of multidisciplinary teams; an individualized approach; interprofessional collaboration; continuous follow-up (in-person and remote); and the existence of support networks with strong rapport and relational skills
Martínez et al. (2016) [19]	Pilot, quasi-experimental study, without a control group	Evaluate the effect of nursing interventions on the perceived burden of informal caregivers of dependent older adults	8	Dyads. The care recipient must be in a state of functional dependence	Implementing a multicomponent intervention, 9 weekly 90-minute home visits (psychoeducational, emotional, and practical) leads to improvements in burden levels and family function: A 27-point reduction in the Zarit scale. A 1,50 point increase in family APGAR
Rodríguez-Lombana & Chaparro-Diaz (2020) [20]	Integrative literature review	Describe social support interventions aimed at family caregivers of people with chronic noncommunicable diseases	19 studies	Family caregivers of people with chronic noncommunicable diseases	Combination of educational components with physical support enhances caregivers’ adherence to interventions and perceived burden reduction. Social support emerges as one of the main protective factors. Nursing interventions based on social support are mostly educational and psychosocial. Burden should be addressed through structured emotional self-care plans, with the nurse serving as the coordinating professional. Interdisciplinary strategies should be implemented; IC must be integrated into the healthcare system

This narrative review was structured around two primary axes: the determinants of informal caregiver burden and the efficacy of nursing interventions. Although a formal risk-of-bias assessment was not performed, rigorous inclusion criteria were applied; the analysis was restricted to studies using validated psychometric instruments and providing comprehensive descriptions of sampling, interventions, and outcome measures.

As a literature-based synthesis, this research required no direct engagement with human subjects nor the collection of personally identifiable information or identifiable data. Therefore, the study relies exclusively on the critical appraisal of secondary data retrieved from peer-reviewed scientific databases.

Discussion of results

Ten articles were included. They were published between 2016 and 2025. This narrative review's findings were categorized into two primary domains: (I) determinants of informal caregiver burden and (II) nursing interventions aimed at the mitigation of such burden.

Determinants of informal caregiver burden

Care Recipient: Related Determinants

The onset of psychological distress and burden among informal caregivers is a multifactorial phenomenon influenced by the demographic, clinical, and psychological profiles of both the caregiver and the care recipient [6]. Evidence indicates a positive correlation between the care recipient's age and their degree of functional dependency, particularly regarding activities of daily living and the incidence of caregiver burden. Caregivers providing assistance with enteral support (eating) and elimination (toileting) exhibit a higher predisposition to elevated burden levels.

Another significant finding links the health of the care recipient to caregiver burden. The care recipient's subjective perception of their health status serves as a significant predictor across all burden domains. Research suggests that the recipient’s perceived quality of life may exert a more substantial influence on caregiver attrition than objective clinical conditions [16].

The 2025 data concerning caregivers of individuals with ostomies reveal profound levels of burden, particularly within the social and emotional dimensions [15]. The burden is precipitated by the complexities of stoma management, including peristomal complications (e.g., prolapse, dehiscence, or irritation), altered body image, and infection risks. These factors intensify the care recipient's physical and psychological requirements, thereby escalating the demands placed upon the informal caregiver [10].

Ultimately, this high level of burden is influenced by the degree of dependency and the physical and mental state of the person being cared for. Among the most frequently implicated factors are reduced social interaction, psychological distress, anxiety associated with the underlying condition, disruption of daily activities, deterioration in sleep quality, and financial pressure [15].

Caregiver: Related Determinants

The impact of caregiver demographics remains a subject of academic debate. Some findings suggest that younger, female caregivers with higher educational attainment are more susceptible to burden. Conversely, other studies contend that lower educational levels correlate with increased burden, citing a deficit in adaptive coping mechanisms and restricted access to health literacy [10].

Further variables contributing to cumulative stress are as follows: The caregiver's familial obligations with conjugal and parental responsibilities are associated with increased burden. The conjunction of domestic, pedagogical, and caregiving duties intensifies physiological and emotional strain [10]. Caregivers with pre-existing morbidities or a self-perception of fragile health demonstrate significantly higher burden vulnerability [6]. Regarding financial determinants, research clarifies that the absolute monthly income is less predictive of burden than the relative financial impact on the household's solvency. Consequently, high levels of burden are reported across diverse income brackets. Social support and physiological well-being are also critical. A significant correlation has been observed between low perceived social support and high burden prevalence [14]. Furthermore, sleep hygiene is a pivotal factor; caregivers reporting fewer than seven hours of sleep per day demonstrate a twofold increase in burden risk. To provide a better understanding of the predictors of informal caregiver burden, Table 2 below summarizes these predictors.

**Table 2:** Predictors of informal caregiver burden

Care recipient's characteristics	Informal caregiver's characteristics
Age (elderly)	Age (young adults)
Functional dependence	Female
Dependence in eating and toileting activities	High/low educational attainment
Self-perceived health status	Duration of care
Presence of ostomies	Marital status
Stoma-related problems	Satisfaction with social support received
	Parenthood
	Self-perceived health status
	Sleep quality

Caregiver burden is a multiversed reality, categorized into three distinct yet interrelated dimensions: emotional, financial, social, and physical burden [13].

Emotional burden: This dimension is characterized by emotional exhaustion and psychological attrition. Precipitated by the chronic and incurable trajectory of the care recipient’s condition, informal caregivers frequently report persistent fatigue and a "overwhelming workload" feeling as primary manifestations of this strain [13].

Financial burden: This arises from the nature of chronic illness, with a prolonged duration, and the requisite need for specialized support materials. While some of the effects of this burden are often mitigated by state subsidy policies, specific contexts, such as that of Portugal and low-income countries, reveal unique stressors. Despite the availability of subsidized or free medical supplies, financial strain persists due to the care recipient’s loss of productivity, the caregiver’s reduced income, or unemployment to perform full-time care [13].

Physical burden: This refers to physical fatigue resulting from continuous vigilance, disrupted sleep, and the demands of nocturnal care. Furthermore, the dual demand of maintaining formal employment to mitigate economic instability while simultaneously fulfilling caregiving duties serves as a significant exacerbating factor for physical depletion [13].

To advance the clinical and scientific understanding of these concepts, it is imperative to determine a sociodemographic and clinical profile of the informal caregiver [15]. Implementing validated psychometric instruments and standardized questionnaires is essential for early burden’s associated risk factors' identification. The systemic mapping of these variables will allow healthcare professionals to provide not a generic support but the development of targeted, evidence-based interventions tailored to the specific multifaceted needs of the caregiving population.

Nursing interventions to mitigate informal caregiver burden

Nurses play a critical role in facilitating the care transition, from in-hospital to the home environment, directly influencing caregiver burden. Effective discharge planning requires a dual approach: on the one hand, it is necessary to provide comprehensive health education regarding postdischarge clinical requirements and, on the other, to equip caregivers with adaptive coping strategies [10].

Prior to intervention design, a nursing assessment is mandatory to evaluate the extent of subjective burden and, at base, self-care deficit among informal caregivers. While specific scales for chronic illness populations remain scarce, established psychometric instruments, such as the Zarit Caregiver Burden Interview [8], the Caregiver Strain Index [21], and the Informal Caregiver Burden Assessment Questionnaire [22], serve as vital diagnostic references. These tools allow nurses to differentiate between individual self-care capacities and faults and the specific requirements for external support [18].

Goeman et al. suggest that psychoeducational Interventions should be prioritized. These represent the gold standard in nursing practice. They integrate educational and psychosocial support, enhancing the caregiver's perceived self-efficacy in patient management [23]. Nevertheless, other tools such as psychotherapy and counseling, multicomponent interventions, and technology-based interventions are also feasible [18].

Psychoeducational interventions and psychotherapy-counseling are among the most commonly used. During sessions, the therapist acts as an agent of change for the caregivers’ social, emotional, and behavioral development. The process redirects the caregiver's acquisition of knowledge and skills to be used in caregiving practice, equipping them with coping, communication, and adaptation strategies [18].

Multicomponent interventions (individual counseling, group work, and telephone support) and technology-based interventions (telephone or computer-based) seem to be the most effective [18]. Nonetheless, the implementation of such measures is under-researched; therefore, the efficacy is still undefined.

Interventions that focus on accurately defining the caregiver’s role and strengthening caregiving skills, promoting the development of strategies for providing quality care, managing family conflicts, effective communication, and relaxation techniques (never forgetting self-care) have proven to be extremely effective and important in mitigating informal caregiver burden. The data suggest that a nursing-implemented educational strategy provided the best results [19].

Cognitive-behavioral therapy is another available tool. It can be used as a strategy to tweak the caregivers’ perceptions of their own negative experiences, correcting dysfunctional interpretations, reducing negative associated emotions, and enhancing personal fulfillment. Additionally, there are other low-cost, low-risk, and easily implementable psychosocial interventions, such as musical therapy, bibliotherapy, and yoga. These have been shown to reduce caregiving-related stress. Written emotional expression is also cited as an effective, accessible, and low-cost intervention [13].

Self-care activities have a profound impact on caregivers' health [16]. Therefore, the authors propose that interventions designed to support caregivers' time management may constitute an effective strategy for mitigating burden. One such example could be a shared care model. In this model, community health professionals (such as primary care nurses) assume certain caregiving tasks. The results were promising. A significant impact on burden with minimal support was shown. 

An interventional program was implemented in 2018, focusing on the well-being of caregivers of people with chronic illnesses. The nursing interventions included educational sessions and workshops; emotional support; guidance on the new role and caregiving skills; promotion of self-care practices; improved techniques for communication and decision-making, and managing family conflicts. After the program, the intervention group found that providing individualized support allowed for a better understanding of the informal caregiver reality in the face of their new role; self-care interventions also reduced anxiety and fear. The caregivers were able to express self-confidence and comfort with their new role, establishing a reciprocal relationship with the care recipient. They expressed feelings of security, confidence, emotional balance, and self-care [17].

Regardless of the chosen method, certain aspects are common to successful and effective interventions, namely: their duration should be at least six to 12 months; being developed by a multidisciplinary/interdisciplinary team relying on interprofessional cooperation to identify actual needs and employing an individualized approach, delivered by trained professionals with continuous follow-up (in person and/or by telephone) [24]. The need to reinforce educational sessions with a physical component is also emphasized, for example, to ensure that caregivers follow the interventions and can apply the content learned in the sessions in practice [20].

Limitations and future directions

The findings of this review are subject to limitations inherent in the narrative methodology. A restricted sample size and the heterogeneity of assessment instruments across papers complicate cross-study comparability. Future research should prioritize the following: (1) robust longitudinal studies to evaluate the long-term impact of nursing interventions on burden markers; (2) instrument validation, the development and validation of specific scales for the Portuguese population to identify "at-risk" profiles; and (3) systemic reorganization, the integration of formal, auditable training programs within hospital admission protocols to ensure early intervention and professional support.

## Conclusions

This narrative review synthesized the most frequent determinants of informal caregiver burden and identified the nursing interventions most effective for burden mitigation. While the necessity for targeted nursing strategies is evident, a notable paucity of research on caregivers’ needs remains. More precisely, a lack of real-world, lived experiences and actual needs of caregivers’ programs. The "care for the caregiver" paradigm must shift from a peripheral consideration to a central tenet of chronic disease management. A holistic approach that positions the caregiver as a partner in care and as an additional recipient of nursing services improved clinical outcomes for the patient and psychological growth for the caregiver.

Ultimately, informal caregiver burden is a predictable and preventable phenomenon. Reorganizing health services to include interdisciplinary support and health education is essential to transform the caregiving experience from one of attrition to one of compassionate resilience and self-care sustainability.
